# Odor Source Localization in Obstacle Regions Using Switching Planning Algorithms with a Switching Framework

**DOI:** 10.3390/s23031140

**Published:** 2023-01-19

**Authors:** Duc-Nhat Luong, Daisuke Kurabayashi

**Affiliations:** Department of Systems and Control Engineering, Tokyo Institute of Technology, 2-12-1 Ookayama, Meguro-ku, Tokyo 152-8552, Japan

**Keywords:** odor source localization, planning algorithm, olfactory robot, algorithm switching

## Abstract

Odor source localization (OSL) robots are essential for safety and rescue teams to overcome the problem of human exposure to hazardous chemical plumes. However, owing to the complicated geometry of environments, it is almost impossible to construct the dispersion model of the odor plume in practical situations to be used for probabilistic odor source search algorithms. Additionally, as time is crucial in OSL tasks, dynamically modifying the robot’s balance of emphasis between exploration and exploitation is desired. In this study, we addressed both the aforementioned problems by simplifying the environment with an obstacle region into multiple sub-environments with different resolutions. Subsequently, a framework was introduced to switch between the Infotaxis and Dijkstra algorithms to navigate the agent and enable it to reach the source swiftly. One algorithm was used to guide the agent in searching for clues about the source location, whereas the other facilitated the active movement of the agent between sub-environments. The proposed algorithm exhibited improvements in terms of success rate and search time. Furthermore, the implementation of the proposed framework on an autonomous mobile robot verified its effectiveness. Improvements were observed in our experiments with a robot when the success rate increased 3.5 times and the average moving steps of the robot were reduced by nearly 35%.

## 1. Introduction

Odor source localization (OSL) tasks are performed to identify the location of leaked hazardous substances or detect the presence of unwanted objects, such as bombs or hazardous chemicals. The source can be determined by tracking the trail of the chemical flow of odor particles diffused into the atmosphere [1]. Using autonomous robots for OSL tasks [2,3,4] can effectively replace humans and trained animals as certain odors may harm living creatures. However, numerous challenges exist in the development and implementation of olfactory robots to perform OSL tasks. The primary challenge is associated with the dispersion of the odor plume into small packets owing to certain unfavorable factors, such as turbulence and gas cavitation near obstacles [5,6]. Additionally, the plume becomes increasingly diluted as it travels away from the source. Most odor search algorithms, including bio-inspired algorithms [7,8,9,10,11,12] and probabilistic or machine learning algorithms [13,14,15,16,17,18], focus on plume finding and tracking. Moreover, most algorithms including the above-mentioned algorithms are primarily designed to be used in obstacle-free environments. Some of them cannot be reproducible in obstacle regions because they require an ideal plume model such as a plume with Gaussian concentration profiles which is nearly impossible to obtain in obstacle regions [19]. In real-world scenarios, the presence of obstacles is most likely to significantly influence airflow [20]; therefore, obstacles cannot be ignored. Although several studies included obstacles in the OSL tasks, they focused on avoiding the obstacles when performing the tasks or considered the obstacles as movable restricted areas. However, studies have not considered the significant impact of obstacles on the airflow of the environment, and this impact has not been used for determining the location of the odor source [21,22,23,24].

We expect that combining some of the conventional odor search algorithms designed to work in obstacle-free environments with obstacle-avoidance functions, such as wall-following, will not address the aforementioned problems. We determined that in the obstacle regions, even the well-established Infotaxis algorithm [13] failed to help the agent in identifying the source at times owing to the occurrence of deadlock situations around the obstacle. This, in turn, prolonged the search time, which should be avoided as time is crucial in OSL tasks.

The objective of this study was to take the advantage of the obstacles rather than considering them as challenges. The presence of obstacles separated the environment into several areas with considerably different characteristics in the airflow distribution, which resulted in variations in odor concentration distribution as well. Based on this, we detected the potential sub-environment containing the source and performed a detailed investigation within that area using the available odor search algorithms. In this study, the Infotaxis algorithm was used as the primary odor search (planning) algorithm owing to its popularity and robustness. This algorithm was combined with a deterministic planning algorithm, namely, the Dijkstra algorithm [25], to enable the agent to move freely between sub-environments. Whenever the agent decided to change the sub-environment to perform the investigation, a switch from Infotaxis to the Dijkstra algorithm was activated. This switching framework aided in completing the OSL tasks in the obstacle regions with improved success rates and reduced search time. Finally, the proposed method was implemented on an autonomous robot equipped with sufficient sensory devices to perform effective OSL tasks in complicated environments.

The components of the algorithm switching framework are described in detail in Section 3. Before that, a review of previous studies in OSL algorithms and problems in OSL are introduced in Section 2. We validate our proposed method by conducting OSL in simulation and in the real world with a robot in Section 4. With the result from conducted experiments, some conclusions are drawn and future works are stated in Section 5.

## 2. Problem Statement

### 2.1. Time-Variant Airflow in the Obstacle Region

**Figure 1:**
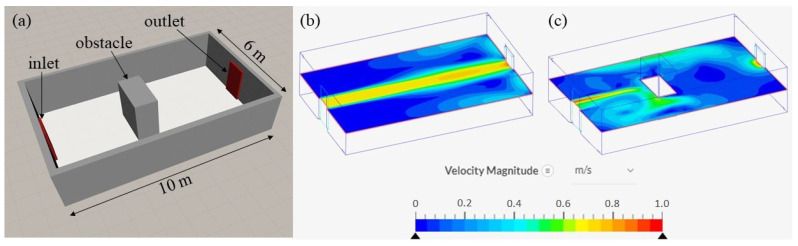
(**a**) Targeted indoor environment with ventilation bounded by walls and (**b**) generated airflow with obstacle-free region and (**c**) generated airflow in obstacle region with a block placed in the middle of the room.

In the indoor environment, airflow is relatively weak as it is generated by one or a few artificial wind sources, such as windows, ventilation systems, and fans. Therefore, the presence of obstacles, including walls, is most likely to significantly influence the airflow. Figure 1 depicts an example of an indoor environment, wherein the impact of a simple obstacle on the airflow is observed. The figure illustrates the airflow velocity distribution at a cross-section plane from one end to the other end of a room with opened doors. The simulation was constructed using SimScale [26], a computational fluid dynamics (CFD) software. We observed considerable variations in the flow directions and speed owing to the displacement of the airflow when contact was made with obstacles.

Most odor-search algorithms are designed for obstacle-free environments to eliminate the aforementioned behaviors of airflow. However, the CFD analysis results indicated that even in a room with an obstacle, a part of the room exhibited the same behaviors of the airflow as observed in obstacle-free environments; this was observed in the half of the room with the upwind direction, where the inlet door was located. If an agent started searching for the source in this half, the OSL task could be simplified to an OSL problem in an obstacle-free environment with a stable airflow field. However, initiating the task from the other half was more challenging because of the considerable variations in the airflow and fluctuations in gas concentration. Consequently, identifying the source starting from the latter half could result in a lower success rate and longer search time.

To take advantage of this difference between the parts of the environment, we decomposed the environment into multiple sub-environments, which could have possibly different airflow behaviors. Subsequently, we used the data from gas sensors and an anemometer to detect the potential sub-environment containing the source, reducing the search time.

### 2.2. Deadlocks in the Probabilistic OSL Algorithm

Among the available probabilistic OSL algorithms, the Infotaxis algorithm proposed by Vergassola et al. [13] is the most popular and highly robust in executing OSL tasks. However, it is designed to be used in obstacle-free environments, which requires the mathematical plume dispersion model. In the presence of obstacles, constructing dispersion models of odor plumes to estimate the odor hit probability is nearly impossible and computationally heavy. Fortunately, Li et al. [27] and Ojeda et al. [28] proposed a model that overcomes the aforementioned problems. In general, an agent can generate short-range estimations of the location of odor plumes using sensory information. These estimations propagate throughout the geometry of the environment, which includes the obstacles, to construct a source location probability map without requiring dispersion models. Subsequently, the Infotaxis algorithm can be used to navigate the agent to the odor source.

**Figure 2:**
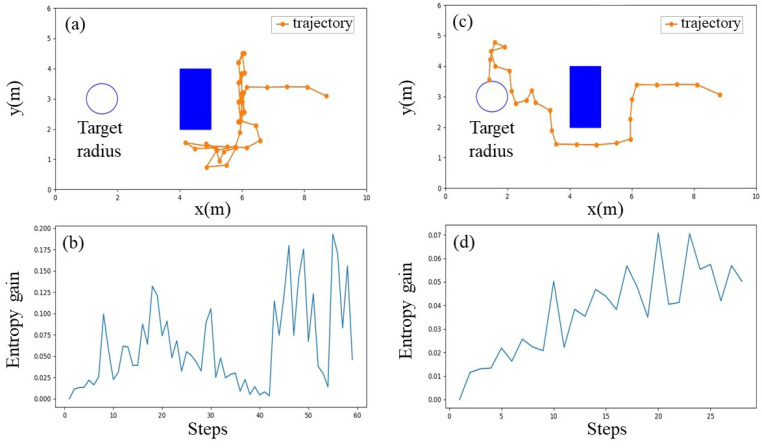
(**a**) Trajectory of a failed search when a deadlock occurs. (**b**) Information entropy gain in the case of deadlock. (**c**) Trajectory of a successful search without a deadlock. (**d**) Information entropy gain in the absence of deadlocks.

However, dealing with obstacles by considering them as restricted areas and constraining the moving space of the agent on the free space is not sufficient. In certain cases, this may reduce the exploration characteristic of the algorithm. Considering the indoor environment depicted in Figure 1 as an example, if the agent is allowed to move following the rule of the Infotaxis algorithm, the chances of the agent moving between two halves of the room through the narrow pathways are limited. In addition to the unstable airflow, this restricted movement of the agent is the main reason for deadlocks in OSL. Deadlock is a situation where the agent is trapped in a certain area and cannot escape the situation to reach the source location. In general, deadlocks occur in areas near the obstacles, as indicated in Figure 2. The figure illustrates the simulation results of OSL tasks in an obstacle region that used the environment depicted in Figure 1 Simulations were performed on GADEN [29], a three-dimensional gas dispersion simulator. All the sensory information was averaged to deal with the vast variations of airflow carrying odor plumes in space. In this case, the presence of the obstacle reduced the success rate by more than half compared to the case with no obstacles. Furthermore, the information entropy gains with respect to the source location distribution probability when deadlocks occurred varied significantly compared to the gradual increase in the entropy gain observed in deadlock-free cases.

Considering that a single planning algorithm is not sufficient for OSL in the obstacle region, we propose using an additional planning algorithm to support the odor search algorithm, namely the Infotaxis algorithm.

### 2.3. Problem Statement

To verify the effectiveness of the proposed method in terms of improving the performance of the Infotaxis algorithm in the obstacle region, both simulation and real-world implementations of the robot were performed. The first planning algorithm, namely the Infotaxis, was used for a step-by-step sampling of the odor around the agent. In this scenario, the source and agent were considered to be in the same sub-environment, and the agent was assumed to eventually reach the source. The second planning algorithm was any applicable graph-search algorithm that could be used by the agent to move easily from one sub-environment to another, wherein the chances of detecting odor and reaching the source were higher.

Typically, an autonomous mobile robot should identify a single releasing source of Ethanol. A robot with a radius of 200 mm was used in this study to perform the OSL tasks in an indoor environment with dimensions of approximately 5000 × 3000 mm. A large single rectangular obstacle relative to the size of the agent was placed in the environment to hamper the agent from completing its task. We decided that our obstacle is rectangular shape because based on the definition of streamlined shape in aerodynamic resistance, in the same fluid conditions (Reynolds number, viscosity, velocity, temperature, etc.), a rectangle shape obstacle generates the widest area of turbulence among basic shapes [30]. In addition, we aim to apply our study for practical use in building environments where we usually encounter obstacles with right angles. Ethanol was released periodically and delivered throughout the environment via artificial airflow generated from two industrial floor fans placed at the location of the source.

The agent used an anemometer (model FT205EV from FT Technologies, Shepperton, UK) [31] to measure the vector of airflow at its location and a set of gas sensors (model MiCS5524 from Adafruit, New York, NY, USA) [32] to measure the Ethanol concentration around it. The agent used the sensory information alone and did not assume or use any dispersion model to make decisions about its actions. The agent reaching the odor source within a certain time limit was considered a successful search; otherwise, it was termed a failed search. The search time and success rate were the two criteria used for the performance evaluation.

In summary, this study aimed to develop an olfactory robot system by considering the following aspects:

1. Performing OSL without a plume dispersion model in the obstacle region;
2. Not relying on only one odor source algorithm;
3. Increasing the success rate and reducing the search time in obstacle regions.

## 3. Materials and Methods

### 3.1. Environment Decomposition

**Figure 3:**
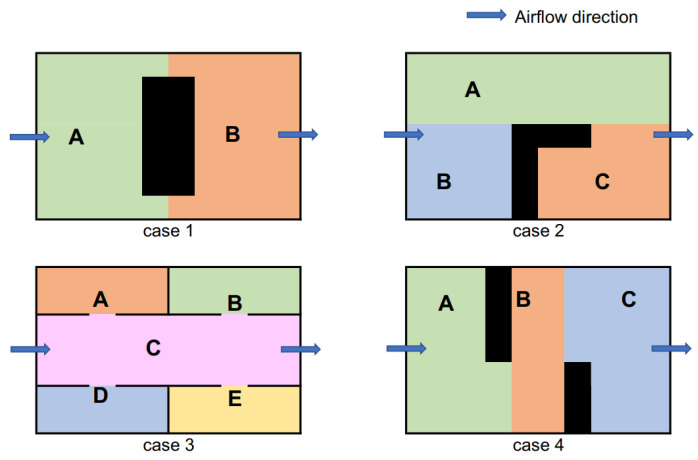
Environments divided into sub-environments (A–E) based on their geometry and airflow direction.

As indicated in Figure 1, a significant difference was observed in the airflow between the two environments on the two sides of the obstacle. In the area of the downwind direction, considerable variations in the airflow directions often resulted in false detection of the direction of odor origin; therefore, the problem of deadlock often occurred in that half. However, if the agent moved to the other half, the source was rapidly reached with nearly no deadlocks. The sub-environments in the upwind direction with respect to the obstacle location exhibited an airflow profile similar to that observed in the obstacle-free environment. Therefore, based on the shape and location of the obstacle with respect to the global airflow, the environment was divided into smaller areas (Figure 3). Black lines and cells represent obstacles and walls, respectively, and the different sub-environments are distinguished by color and alphabets.

In this study, we considered the environment depicted in case 1 (Figure 3) as the target and decomposed it into two sub-environments, A and B. Despite the simplicity, a previous study reported that even an expert OSL task performer, such as the silkworm moth, failed to perform well in a similar environmental setup [33]. Regarding the obstacle position, any position can be considered feasible for experiments. We considered only the center position because we expect that the position of the obstacle in the upwind direction where it blocks all the straightforward movement of odor plumes from the source is the most challenging. After decomposing the environment, we identified a framework that enabled the agent to decide whether its movement should be step-by-step in terms of performing the sequence of odor sampling and updating the source location probability map or whether the agent should jump to the more promising sub-environment to perform the same sequence of actions.

### 3.2. Switching between Planning Algorithms

In the Infotaxis algorithm, an odor plume tracing agent attempted to localize the source based on movements to reduce the information entropy of the source location probability. At any location, for each action, *a* in the set of available actions in the Infotaxis algorithm, $\left(A^{\prime }=\left\{\mathrm{move}\;\mathrm{left},\mathrm{move}\;\mathrm{right},\mathrm{move}\;\mathrm{forward},\mathrm{move}\;\mathrm{backwards}\right\}\right)$, the agent selected the best action $a^{*}$ which resulted in the maximum expected information entropy gain ΔS, calculated using the Kullback–Leibler divergence [34] as

(1) $$a^{*}=\underset{a\in A^{\prime }}{\mathrm{argmax}}E\left[\Delta S\left(a\right)\right]$$

However, this strategy served as a local planner, which navigated the agent only to the next location near the boundary despite the availability of a source location probability map of the entire environment. This implied that even if certain areas intuitively exhibited a high certainty of containing the source, the agent attempted to move in a small unit step size to gain more information about its surroundings. However, time is essential during rescue and safety missions. Moreover, certain situations may require the agent to break the rules of movement of the Infotaxis algorithm and move directly to the areas that exhibit a high potential of containing the source. Based on our experience of designing and running OSL robots, we determined that the search time is significantly dominated by the moving time of the robot rather than the time required for odor sampling and information processing. Therefore, reducing the number of moving steps is crucial.

Maintaining the strength of the Infotaxis algorithm in dealing with the exploration and exploration dilemma [35] in OSL, we dynamically changed the planning algorithm of the agent to facilitate its jumping to promising areas. Here, the promising area indicated the sub-environment with the highest probability of containing the odor source. The environment decomposition generated two grid maps with two resolutions. The higher resolution (smaller cell size) was referred to as the local resolution, whereas the lower resolution was considered the global resolution. Each cell of the local resolution comprised a probability of source existence in that cell, with $c_{ij}\in C$ as the cell in the *i*-th row and *j*-th column of the grid map C with local resolution. We considered an environment with *M* rows and *N* columns, which could be decomposed into *K* sub-environments. Here, a sub-environment $E_{k}$ exhibiting the probability of containing the source was calculated by summing the probability of all local cells within that sub-environment, as follows:

(2) $$p\left(E_{k}\right)=\sum_{c_{ij}\in E_{k}}p\left(c_{ij}\right)$$

where:

(3) $$\sum_{k=0}^{K}p\left(E_{k}\right)=\sum_{i=0}^{N}\sum_{j=0}^{M}p\left(c_{ij}\right)=1$$

**Figure 4:**
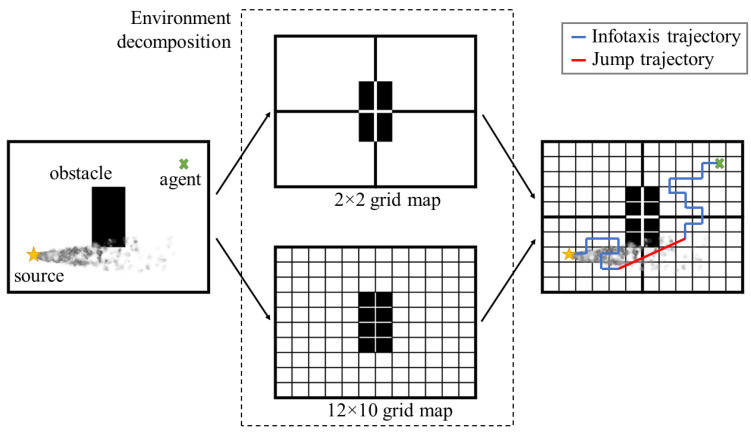
Multiple resolution grid maps are used for multiple planning algorithms in the odor source localization (OSL) task.

**Figure 5:**
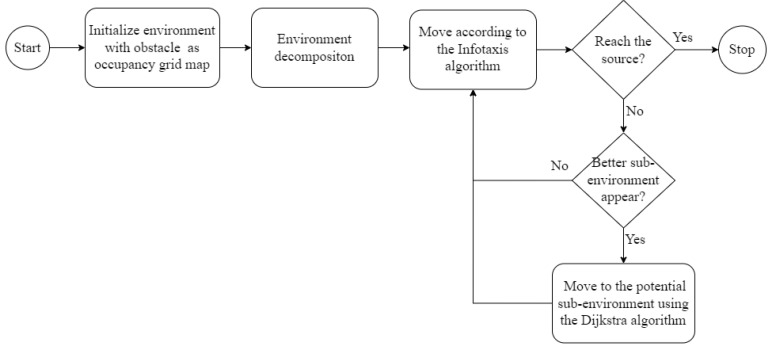
The workflow of the planning algorithms switching.

Figure 4 depicts the jump as a red line, which was generated using any deterministic graph search planning algorithm. In this study, we adopted the Dijkstra algorithm [25], which enabled the application of the jump in the obstacle region as the shortest collision-free path and not merely a direct straight connection between the current location of the agent and the potential target of the preferred sub-environment. Consider the grid map with the unit resolution, the vertices for planning using Dijkstra’s algorithm are defined as the center points of each free cell. Dijkstra’s path is then constructed as a set of connection lines between adjacent centers. When a switch to Dijkstra’s algorithm is activated, the single source of the algorithm is defined as the cell having the highest probability of containing the odor source within the preferred sub-environment. After the jump, the planning algorithm switched back to Infotaxis, and the agent continued to detect the presence of odor plumes in the new area. Figure 5 presents a flowchart of the proposed switching framework of the planning algorithm.

Among *K* number of dividend sub-environments, denote $E_{k^{*}}$ as the one that has the highest sum of probability of containing the source in each cell within it, we have:

(4) $$k^{*}=\underset{k\in K}{\mathrm{argmax}}p\left(E_{k}\right)$$

The jumping can be expressed as the output of the function of the Dijkstra planning algorithm f:C→A. This function returns the best (shortest) moving action for the agent to move to any given destination cell $c_{ij}$ in the grid map C. A denotes the set of all actions that the agent can perform to reach each cell which includes the Infotaxis algorithm actions set $A^{\prime }\subset A$. Therefore, our framework for finding the best action $a^{*}$ for the agent at any step can be represented as:

(5) $$a^{*}=\left\{\begin{array}{cc} f[\underset{c_{ij}\in E_{k^{*}}}{\mathrm{argmax}}p\left(c_{ij}\right)] & \mathrm{if}\;p\left(E_{k^{*}}\right)>\rho \frac{1}{K},\\ \underset{a\in A^{\prime }}{\mathrm{argmax}}E\left[\Delta S\left(a\right)\right] & \mathrm{otherwise}. \end{array}\right.$$

Here, the condition of the first term indicates that a jump should be performed only when the sub-environment exhibits the highest probability of standing out in comparison with the initial possibility of each sub-environment in a uniform distribution $p_{i}\left(E_{k}\right)=\frac{1}{K}$. The stand-out coefficient ρ is crucial for deciding the timing and frequency of the jump during the search. A detailed explanation is presented in Section 4.2. The pseudocode of the combination between two algorithms for the OSL task is described in the Algorithm 1.
**Algorithm 1** Algorithms switching framework     **Data:** *agent, source*                           ▹ Location of agent and odor source     *      gridMap, obstacle*                        ▹ Given environmental components1:**procedure**OSL2:    sourceFound← false3:    **for all** $c_{ij}$ in gridMap **do**4:        $p(c_{ij})\leftarrow 1/$size(gridMap)                           ▹ Initialize uniform distribution5:    **end for**6:    $L_{env}\leftarrow$ decompose(gridMap,obstacle)                                   ▹$L_{env}=\left\{E_{1},E_{2},\cdots ,E_{K}\right\}$7:    **while** *sourceFound =* false **do**8:        $E_{k^{*}}\leftarrow$ max($L_{env}$)9:        **if** agent in $E_{k^{*}}$ **then**                                                  ▹ Switch to Infotaxis algorithm10:           $action\leftarrow \underset{a\in A^{\prime }}{\mathrm{argmax}}E\left[\Delta S\left(a\right)\right]$11:           agent← move(*agent, action*)12:        **else**                                                                             ▹ Switch to Dijkstra algorithm13:           $jumpTarget\leftarrow \underset{c_{ij}\in E_{k^{*}}}{\mathrm{argmax}}p\left(c_{ij}\right)$14:           path← dijkstra*(agent, jumpTarget)*15:           agent← move(*agent, path*)16:        **end if**17:        sensorValue← sampling()18:        gridMap← update(agent,sensorValue,gridMap)19:        **if** ∥agent−source∥<targetRadii **then**20:           sourceFound← true.21:        **end if**22:    **end while**23:**end procedure**

## 4. Results

### 4.1. Verification of the OSL with Environment Decomposition

**Figure 6:**
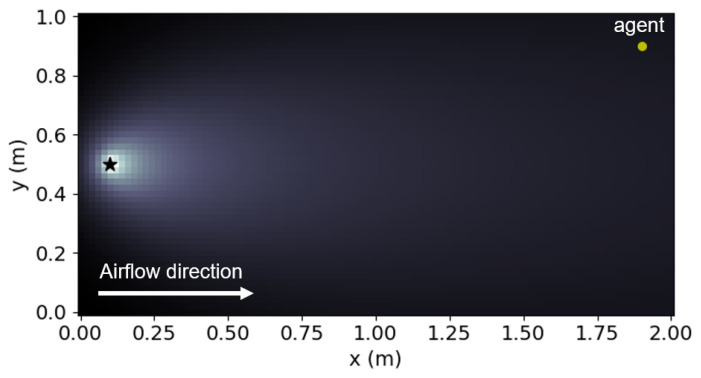
Plume profile for testing based on the airflow direction. The star indicates odor source location.

**Figure 7:**
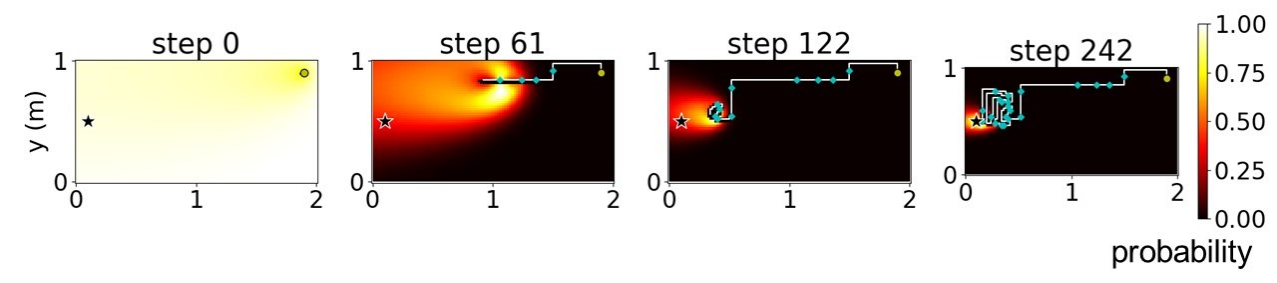
Timeline of a typical odor source localization (OSL) search using the Infotaxis algorithm without environment decomposition (Number of sub-environments (K)=1). The star indicates odor source location.

**Figure 8:**
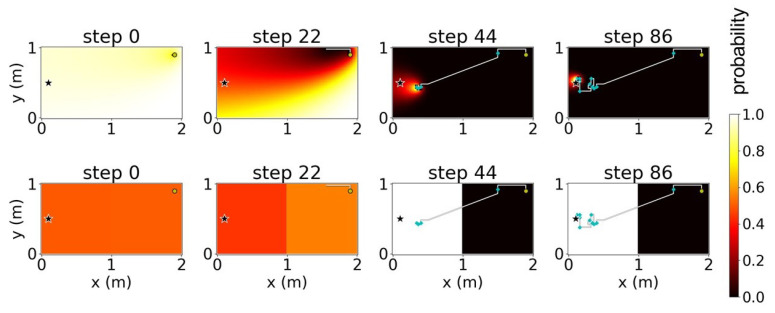
Timeline of a search using environment decomposition with the number of sub-environments (K)=2 and stand-out coefficient (ρ)=1. The star indicates odor source location.

**Figure 9:**
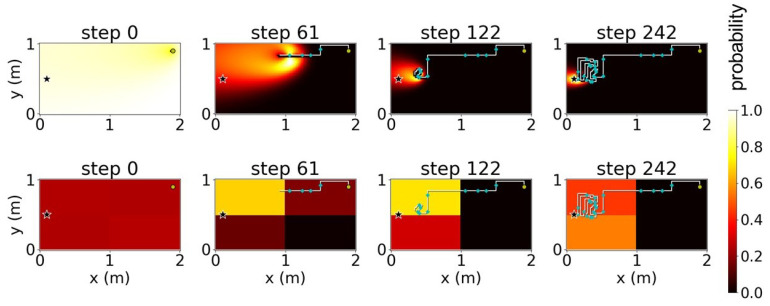
Timeline of a search using environment decomposition with the number of sub-environments (K)=4 and stand-out coefficient (ρ)=2. The star indicates odor source location.

**Figure 10:**
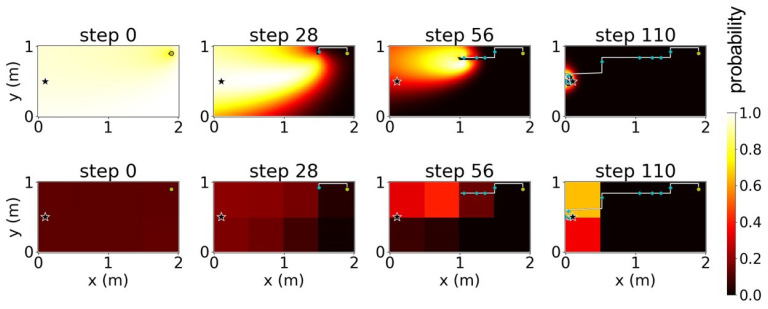
Timeline of a search using environment decomposition with the number of sub-environments (K)=8 and stand-out coefficient (ρ)=4. The star indicates odor source location.

We verified the proposed method using simulations of a free environment with dimensions 2 × 1 m and a pre-defined uniform plume dispersion model (Figure 6); the star symbol in Figure 6 indicates the true location of the odor source. We attempted to divide this environment into four cases. With *K* as the number of sub-environments obtained after the decomposition, we considered four cases of K∈{1,2,4,8} where K=1 indicates the absence of decomposition. Figure 7, Figure 8, Figure 9 and Figure 10 illustrate one successful search of each case. Figure 7 and the upper rows in Figure 8, Figure 9 and Figure 10 depict the timeline of the search in a grid map of 100 by 50 cells, where the color of each cell indicates the probability of the source existing in the cell; the lower rows indicate the change in the total probability of the source location of all cells within each sub-environment. The white lines denote the trajectories of the agent and its path to the source, whereas the cyan points depict the event of detecting the odor plume.

**Figure 11:**
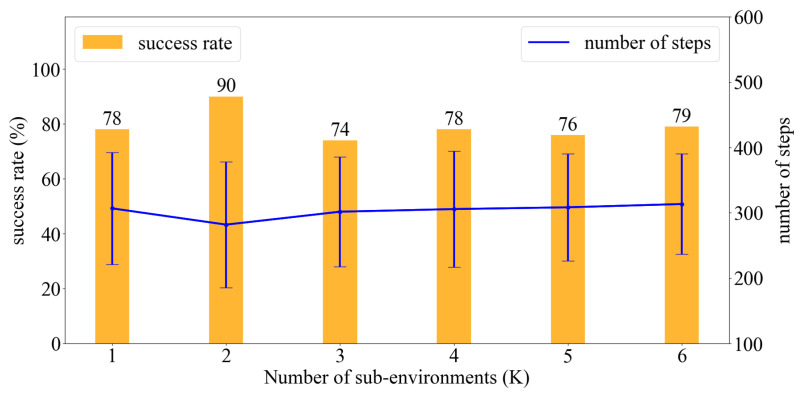
Calculation of success rates and average steps for a varying number of sub-environments.

To verify whether the proposed method improved the quality of a search, we maintained a ratio of ρ/K=0.5 for each case of *K*, extended to K=32. We performed 100 trials and recorded the success rate along with the average moving steps of the agent. A failed search in the experiment was defined as a search where the agent could not reach the source within 400 steps. Figure 11 depicts the obtained experimental results.

Although no apparent improvement was observed when the environment was divided into four or more sub-environments, dividing the environment into half significantly increased the success rate from 78 to 90% and reduced nearly 20% of the moving steps. In other words, the moving time for the agent was considerably reduced. Moreover, the obstacle region in case 1 (Figure 3) indicated that decomposing the environment into half was recommended. Based on this result, we performed an actual OSL task using an autonomous robot in a similar environment to verify whether improvement observed in the obstacle-free environment can be achieved. In addition to this, we analyzed the role of the stand-out coefficient ρ in the frequency of jumping action.

### 4.2. Jumping Frequency

**Figure 12:**
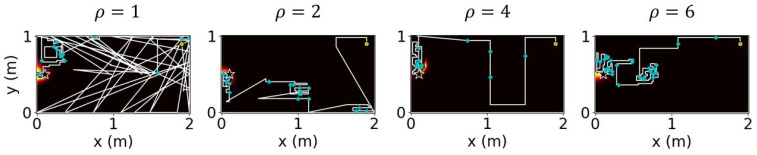
Trajectories of the agent with different stand-out coefficients. The star indicates odor source location.

**Figure 13:**
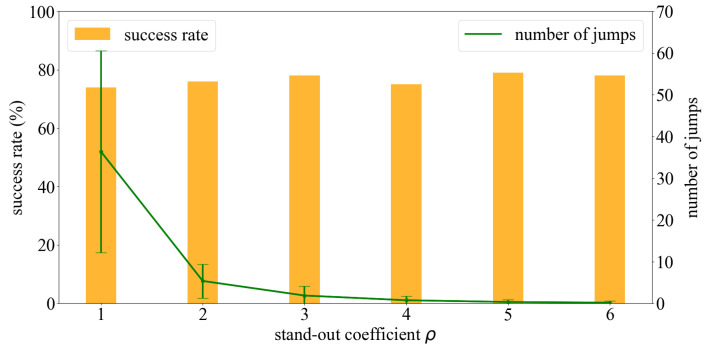
With the same K=8, different stand-out coefficients result in a similar success rate but the number of jumps changes significantly.

The agent jumped to a sub-environment whenever the highest probability of containing the source was exhibited by that sub-environment. However, with this condition alone, the agent jumped exceedingly often owing to slight changes in the probabilistic value of each cell in each step. Frequent jumps implied that the search involved heavy exploitation, which in turn reduced odor sampling and increased the search time. Considering the case of K=8 as the target, where each sub-environment exhibited the initial possibility of containing the source $p_{i}=1/8$, we examined the change in success rate, average steps, and the average number of jumps with different stand-out coefficients. Figure 12 depicts the obtained results, wherein we observed that the smaller the stand-out coefficient, the more chaotic the trajectory. This is not desirable in real-world applications despite the improvements in success rate and the number of moving steps. With a fixed number of *K*, different stand-out coefficients resulted in similar success rates and average moving steps; however, the number of steps decreased drastically with the increase in the value of the stand-out coefficient. Figure 13 illustrates the results when the environment was divided into eight sub-environments and the stand-out coefficient was varied from 1 to 6. We performed 100 simulations for each stand-out coefficient and determined that a fixed ratio of ρ/K=0.5 was the most effective for each case of *K*. This was because the number of jumps often required approximately 0.4% of the total moving steps which are reasonable.

The shape and location of obstacles also affect indirectly the frequency of switching. The more turbulent the flow created by the obstacle, the more uncertainty in the estimation of source location and therefore the more uncertainty in the gas source location probability distribution. The distribution will have multiple local maxima instead of gradual change. This requires the agent to switch algorithms frequently to investigate local maxima quickly.

### 4.3. Result of Algorithm Switching in the Real World with a Robot

**Figure 14:**
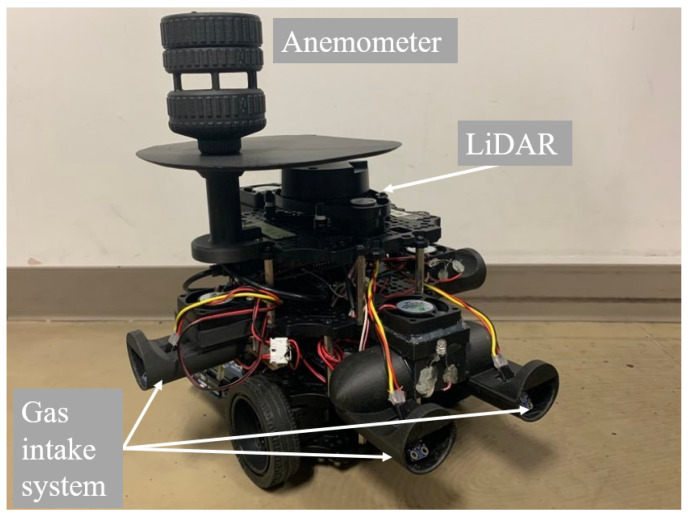
Bombyxbot3, our designed olfactory robot.

To address the problems of OSL in the obstacle region, we verified the performance of the proposed method on a mobile robot. Inspired by the *Bombyx mori* [36], our in-house olfactory robot Bomboyxbot3 was built on the base of the TurtleBot3 Burger [37]. The robot was equipped with an anemometer, five gas sensors in four directions, and a Light Detection and Ranging (LiDAR) sensor. Given the characteristics of slow response time and slow recovery time, it is not good to determine the presence of alcohol by simply setting a threshold value for the sensor output. Thus, we applied an autoregressive with exogenous input (ARX) model as the inverse model to the sensor output to estimate the presence of alcohol in the air [38]. To deal with the unpredictable movement of airflow, we use the averaged gas measurement from alcohol sensors. In addition, we equipped sucking fans behind each sensor so that we can capture the odor plume in a certain volume around the agent. Figure 14 depicts a snapshot of the designed Bomboyxbot3.

**Figure 15:**
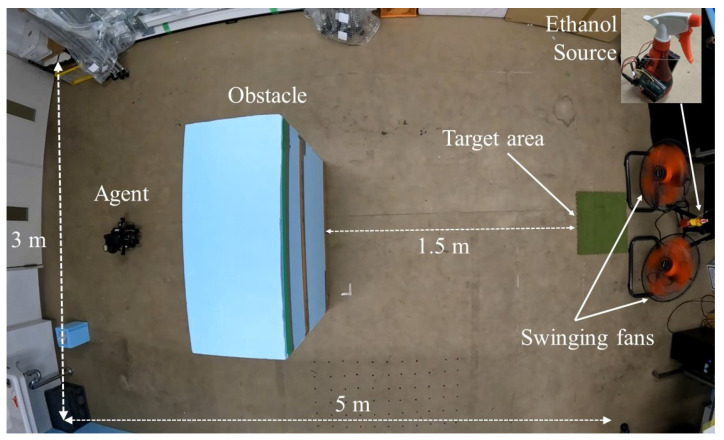
Setup of the experimental environment.

Ethanol 50% was used as the leaked substance in the experimental setup. Figure 15 depicts an artificial leaked Ethanol gas source created using an automatic hand spray. The Ethanol was sprayed periodically via a servo motor, which was used to control the release rate and spraying ranges. During the experiments, the source was placed at a height of 600 mm, and Ethanol was released at 0.35 Hz with a flow rate of 0.08 mL/s. The dimensions of the tested indoor environment were 3300 × 5000 mm with no open windows. Furthermore, the ventilation system of the room was turned off. The average room temperature was around 27 °C. Two industrial floor fans were placed at the source location (Figure 15). The fans were 550 mm high, and an obstacle with dimensions 450 × 1000 × 900 mm (W × L × H) was placed in the middle of the room. Figure 15 indicates the locations of the gas source, fans, and obstacle. In terms of the search time limitation, a search that lasted more than 800 s during the experiments was considered a failure. The resolution of the probability maps was set to 0.5 m. The proposed method was implemented considering two cases of using switching between algorithms. The two cases were:

1. Find the source with Infotaxis algorithm without switching;
2. Find the source with algorithm switching.

**Figure 16:**
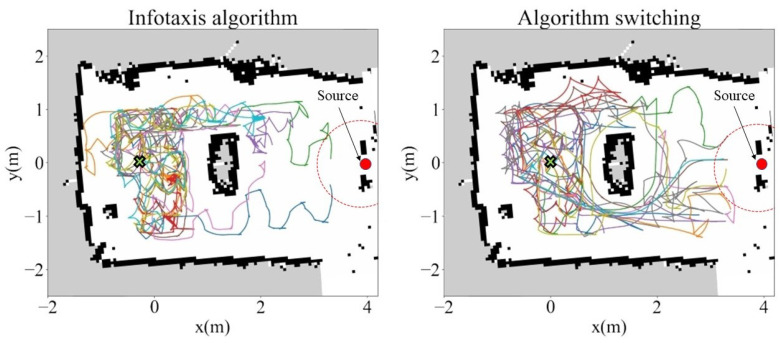
Trajectories of the robot in two cases, with ten trials conducted for each case. The dashed red lines indicate the target radii.

**Figure 17:**
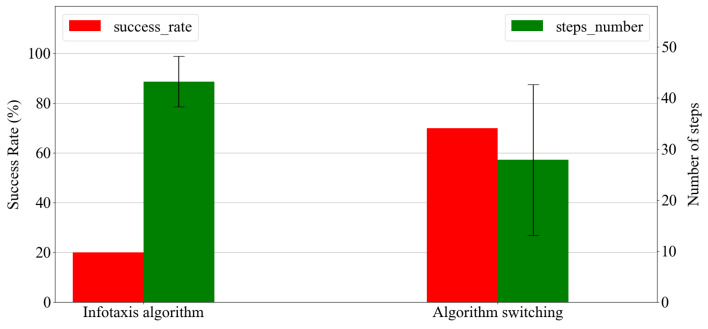
Success rate and search time for the two cases.

Ten trials were performed for each case and all trajectories were recorded, as illustrated in Figure 16. The trajectories indicated that without the switching between algorithms, deadlocking in the obstacle region occurred in several trials. Moreover, the agent spent a long time moving behind the obstacle. This was attributed to the lack of gas detection and the large range variation in airflow directions recorded with the anemometer in the area behind the obstacle. However, a comparison of success rates and average search time was performed in each case to observe the differences; the obtained results are plotted in Figure 17.

As indicated in Figure 17, the addition of a simple obstacle significantly reduced the effectiveness of the Infotaxis algorithm. When the robot started from the center in the obstacle region without using the proposed method of switching algorithm, the success rates were only 20%; however, implementing the algorithm switching framework increased the corresponding success rates to 70%. Regarding the moving steps performed by the agent, by using the proposed method the average number of steps was reduced by nearly 35% from 43.2 to 27.9 steps. The results validated that the proposed method improved the success rate of the OSL tasks in the obstacle region and it also reduced the redundant movements of the agent. Therefore, our research objectives were satisfied.

## 5. Discussion

In this study, we performed OSL tasks using an autonomous robot in obstacle regions considering different planning algorithms. We successfully switched back and forth between the Infotaxis and Dijkstra algorithms using an algorithm-switching framework, which significantly improved the execution of the examined OSL task in the obstacle regions. Furthermore, under identical environmental conditions, the average search time was shorter and the success rate was higher when the proposed method was implemented as compared to the results observed when our method was not used.

Therefore, in accordance with the problem statement and objective of this study, we successfully constructed a switching algorithm framework and assessed its performance using autonomous robot experiments as an engineering application. However, although the running time of the OSL tasks was slightly long, a considerable amount of this time involved the robot planning a path from the current location to its neighboring locations and its movement. In the future, we intend to use another robot platform, such as a holonomic robot, to reduce the time required for navigation.

Another problem caused by the time-consuming movement between destinations (cells) was the wastage of gas detection. As gas detections were considered only when the robot arrived at the center of the next cell, all detections during the movement were ignored. This was a significant difference between real-world experiments and simulations, wherein the resolution of the grid map was high. Typically, the frequency of gas sampling in a high-resolution grid map is fast and can be considered continuous. To address the aforementioned problem, future investigations should focus on combining the cognitive–reactive algorithm with a probabilistic algorithm. This study considered the Infotaxis algorithm as the primary odor search algorithm owing to its popularity and robustness. However, any algorithm can be used and integrated for operating the switching framework. Future studies could focus on this aspect to improve the framework based on our study findings.

## Abbreviations

The following abbreviations are used in this manuscript:

OSL	Odor Source Localization
CFD	Computational Fluid Dynamics
LiDAR	Light Detection and Ranging
